# Evaluating the levels of CSF and serum factors in ALS

**DOI:** 10.1002/brb3.637

**Published:** 2017-02-19

**Authors:** Jie Guo, Xuan Yang, Lina Gao, Dawei Zang

**Affiliations:** ^1^ Department of NeurologyTianjin First Center HospitalTianjin Medical UniversityTianjinChina

**Keywords:** amyotrophic lateral sclerosis, chemokines, cytokines, growth factors

## Abstract

**Objectives:**

The aim of this study was to identify CSF and serum factors as biomarkers that may aid in distinguishing ALS patients from control subjects and predicting ALS progression as well as prognosis.

**Methods:**

Serum and CSF samples from 105 patients with ALS and 56 control subjects were analyzed for 13 factors using ELISA. The revised ALS functional rating scale (ALSFRS‐r) was used to evaluate the overall functional status of ALS patients, and we also followed up with ALS patients either by phone or with clinic visits for five years after enrollment in this study. Finally, we examined the correlations between factor levels and various clinical parameters and evaluated the predictive value for prognosis through a multivariate statistic model.

**Results:**

A total of eight factors were obviously elevated in CSF, and twelve markers were increased in serum. In the correlation analyses, there were trends toward higher bFGF, VEGF, MIP‐1α levels in ALS with a longer disease duration and slower disease progression in both CSF and serum. Higher MCP‐1 levels were associated with worse disease severity and faster progression, and the IFN‐γ levels were positively associated with disease progression in either CSF or serum. Finally, a better prognosis was observed with higher levels bFGF in CSF and VEGF in CSF and serum; conversely, patients with higher levels of IFN‐γ in the CSF had shorter overall survival.

**Conclusions:**

We demonstrated that a factor profile of ALS patients is distinct from control subjects and may be useful in clinical practice and therapeutic trials.

## 1 Introduction

Amyotrophic lateral sclerosis (ALS) is a fatal neurodegenerative disorder that affects motor neurons in the brain and spinal cord leading to paralysis and eventually death (Hardiman, Van den Berg, & Kiernan, 2011). There have been various ALS biomarker studies published with more ongoing, with special attention focused on identifying biomarkers in CSF or serum. Most of these studies have examined changes of individual biomarkers that might distinguish patients with ALS from healthy control subjects (Lu et al., 2015; Takahashi et al., 2015), but it is likely more appropriate to identify panels of biomarkers rather than focusing on a single factor to increase their specificity to ALS. Some studies were limited by the number of samples in the analysis (Turner et al., 2013), and some chose a different control population into the study (Tateishi et al., 2010). In addition, a panel of biomarkers has been detected in either the CSF or serum in some recent studies, but they were either more concerned with a diagnosis rather than a clinically relevant prognosis (Gray et al., 2015; Kuhle et al., 2009; Lawton et al., 2012) or only measuring factors either the CSF or serum (Ehrhart et al., 2015; Mitchell et al., 2009).

We previously reported that several cytokines are elevated in the CSF and serum of ALS patients (Gao, Zhou, Cai, Gong, & Zang, 2014; Gong, Gao, Guo, Lu, & Zang, 2015; Liu, Gao, & Zang, 2015; Yang, Gao, Wu, Zhang, & Zang, 2016). Here we expand on our earlier results by measuring a panel of factors in the CSF and serum from a larger group of ALS patients to intuitively and comprehensively show the differences in the factor levels between ALS patients and control subjects. Then, we further analyzed the associations between the factor levels and clinical parameters to identify biomarkers related to disease progression and prognosis. Finding reliable biomarkers in ALS is valuable to provide an early diagnosis and the preliminary basis for disease pathogenesis in future studies.

## 2 Materials and Methods

### 2.1 Subjects

This study was approved by the Clinical Experimentation Committee of Human of Tianjin First Center Hospital (2014022S). All the participants in the study provided written informed consent prior to enrollment. A total of 105 patients with sporadic ALS and 56 control subjects evaluated in the neurological wards of Tianjin First Center Hospital between April 2006 and June 2015 were enrolled in the study. Eligible patients were diagnosed by experienced neurologists according to revised E1 Escorial criteria (Brooks, Miller, Swash, & Munsat, 2000), which included clinically definite or probable ALS. ALS patients with any medical condition related to motor neuron dysfunction or who were taking any medicine were excluded. Disease duration was defined as the interval between onset of symptoms and diagnosis. The revised ALS functional rating scale (ALSFRS‐r) is a well‐established scale to evaluate overall function of ALS patients and is scored from 0 to 48, with lower scores indicating poorer function (Gladman, Dehaan, Pinto, Geerts, & Zinman, 2014). The disease progression rate (DPR) was calculated using the following formula: DPR = (48 – ALSFRS‐r score at time of diagnosis)/disease duration (months). We also followed up ALS patients either by phone or with clinic visits for 5 years after enrollment in this study, and the primary endpoint was death. Survival time was defined as the interval between the time of diagnosis and death from confirmed ALS‐related complications. Fifty‐six control subjects were diagnosed with tension‐type headache (*n *= 34), hypokalemic paralysis (*n *= 8), cerebrospinal fluid leakage (*n *= 4), or low intracranial pressure (*n *= 10). However, we excluded controls with any life‐threatening disease or who were taking any medicine. The demographic features, including age, gender, and body mass index (BMI), did not show significant diversity between the ALS patients and control subjects (*p *>* *.05). The demographic and clinical characteristics are summarized in Table 1.

**Table 1:** Demographic and clinical features of patients with sporadic ALS and control subjects

Subjects	ALS	Con
Patients	105	56
Clinically definite/probable	72/33	–
Gender (M/F)	56/49	31/25
Age at examination (years)	58.04 ± 10.33	56.98 ± 11.41
BMI	24.33 ± 3.20	25.61 ± 5.17
Sample (CSF/S)	81/105	56/56
Site of onset		
Limb	67	–
Bulbar	23	–
Both	15	–
Duration (months)	30.70 ± 29.70	–
ALSFRS‐r score	33.35 ± 7.20	–
DPR	0.82 ± 0.67	–

M, Male; F, Female; BMI, Body mass index; CSF, Cerebrospinal fluid; S, Serum; ALSFRS‐r, Revised amyotrophic lateral sclerosis functional rating scale; DPR, Disease progression rate.

### 2.2 Serum and CSF samples collection

Blood samples were available from all the patients by venipuncture and centrifuged immediately to isolate serum. The supernatant was stored at −80°C until further use. CSF samples were simultaneously obtained by lumbar puncture from 81 ALS patients and 56 controls and quickly centrifuged prior to storage of the supernatant at −80°C. Blood and CSF were collected between 8 AM and 12 PM to limit circadian effects.

### 2.3 Immunoassays

The serum and CSF samples were detected for the following 13 factors: IL‐2 (R&D, D2050), IL‐6 (R&D, D6050), IL‐10 (R&D, D1000B), IL‐15 (R&D, D1500), IL‐17 (R&D, D1700), G‐CSF (R&D, DCS50), GM‐CSF (R&D, DGM00), bFGF (R&D, DFB50), VEGF (R&D, DVE00), MIP‐1α (R&D, DMA00), MIP‐1β (R&D, DMB00), MCP‐1 (R&D, DCP00), and IFN‐γ (R&D, DIF50). Every factor was assayed using ELISA kits and conducted per the manufacturer's protocols. All the marker measurements were performed by blinded independent investigators to avoid subjective bias.

### 2.4 Statistical analysis

Analyses were conducted using GraphPad Prism Version 5.0 (RRID:SCR_002798) and SPSS Statistics version 17.0 (RRID:SCR_002865). Continuous demographic and clinical data were described as the means ± standard deviation (SD) in the tables. Either a two sample *t*‐test or Mann–Whitney *U*‐test was used to test the differences in factor levels between the groups. Correlations between the factor levels and various clinical parameters were assessed by either Pearson or Spearman correlation analysis. Univariate and multivariate survival analyses were performed using Cox proportional hazard models to evaluate the effects of several independent variables on survival, including demographic and clinical data. The univariate survival analysis selected covariates into the multivariate Cox proportional hazard model using a low threshold of *p *<* *.2 to include variables with potential or possible effects. Risks were expressed as the hazard ratio (HR) and 95% CI. The significant level was set at *p *<* *.05.

## 3 Results

### 3.1 Factors level in CSF and serum

First, to identify factors that are obviously different between ALS patients and control subjects, we detected 13 factors in serum and CSF samples using ELISA. The analytical results of the factors are detailed in Table 2. In the CSF, IL‐15, IL‐17, bFGF, VEGF, MIP‐1α, MIP‐1β, MCP‐1, and IFN‐γ were significantly elevated in ALS patients compared with control subjects. In contrast, there were more factors notably altered in serum; among these, IL‐2, IL‐6, IL‐10, IL‐15, IL‐17, G‐CSF, GM‐CSF, bFGF, VEGF, MIP‐1α, MCP‐1, and IFN‐γ levels were strongly increased in ALS patients. The levels of other factors showed no significant differences between the two groups.

**Table 2:** Biomarker levels in the CSF and serum of patients with ALS and control subjects

Marker	CSF (pg/ml)			Serum (pg/ml)		
	ALS	Con	*p*	ALS	Con	*p*
IL‐2	414.17 ± 63.11	393.29 ± 68.47	0.0695	571.86 ± 111.82	370.09 ± 95.46	**<0.0001**
IL‐6	124.25 ± 27.35	119.56 ± 25.88	0.3153	211.83 ± 69.96	114.05 ± 32.26	**<0.0001**
IL‐10	227.74 ± 39.95	218.55 ± 54.65	0.2572	457.86 ± 117.73	214.92 ± 78.39	**<0.0001**
IL‐15	251.17 ± 30.16	155.77 ± 33.64	**<0.0001**	420.91 ± 74.10	184.48 ± 43.00	**<0.0001**
IL‐17	21.93 ± 3.86	11.27 ± 2.62	**<0.0001**	26.06 ± 8.68	12.96 ± 3.33	**<0.0001**
G‐CSF	187.66 ± 78.91	166.86 ± 48.61	0.0816	425.67 ± 136.25	161.27 ± 60.54	**<0.0001**
GM‐CSF	175.72 ± 32.02	169.32 ± 35.22	0.2712	252.86 ± 48.64	140.62 ± 34.03	**<0.0001**
bFGF	327.07 ± 37.55	240.74 ± 37.36	**<0.0001**	423.72 ± 65.07	278.09 ± 29.24	**<0.0001**
VEGF	717.92 ± 94.30	539.40 ± 87.16	**<0.0001**	1430.85 ± 339.73	569.79 ± 90.73	**<0.0001**
MIP‐1α	300.15 ± 53.04	186.23 ± 56.86	**<0.0001**	353.46 ± 66.59	183.74 ± 41.98	**<0.0001**
MIP‐1β	513.65 ± 57.04	349.84 ± 53.30	**<0.0001**	566.40 ± 67.93	564.31 ± 103.99	0.8777
MCP‐1	330.08 ± 86.47	299.63 ± 23.59	**0.0113**	549.89 ± 131.26	335.39 ± 77.67	**<0.0001**
IFN‐γ	371.53 ± 34.54	184.30 ± 29.11	**<0.0001**	297.33 ± 31.79	133.55 ± 19.60	**<0.0001**

The bold values indicates that the results are statistically significant.

### 3.2 Correlations between factors level and various clinical parameters

**Figure 1:**
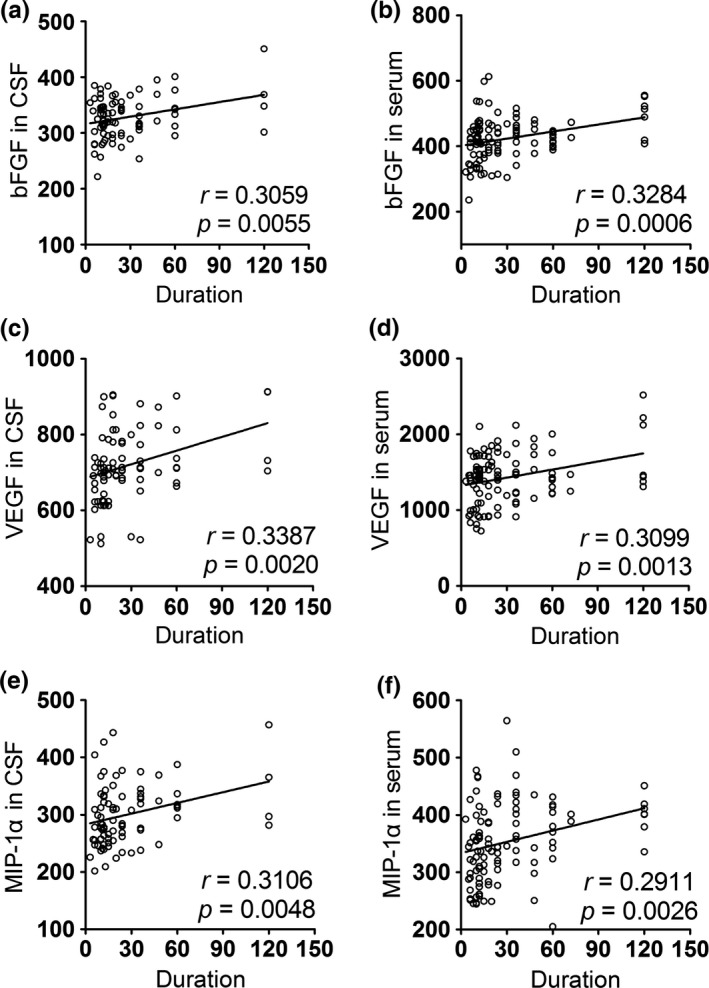
Correlations between the level of biomarkers in CSF/serum and duration. (a) The level of bFGF in CSF was positively correlated with the duration of ALS patients. (b) The correlation between the bFGF level in serum and duration was positive. (c) The level of VEGF in CSF was positively correlated with the duration of ALS patients. (d) The correlation between the VEGF level in serum and duration was positive. (e) The level of MIP‐1α in CSF was positively correlated with the duration of ALS patients. (f) The correlation between the MIP‐1α level in serum and duration was positive

**Figure 2:**
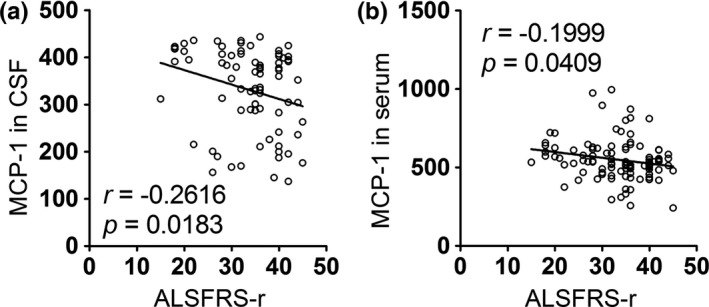
Correlations between the level of biomarkers in CSF/serum and the ALSFRS‐r score. (a) The level of MCP‐1 in CSF was negatively correlated with the ALSFRS‐r score of ALS patients. (b) The correlation between the level of MCP‐1 in serum and the ALSFRS‐r score was negative

**Figure 3:**
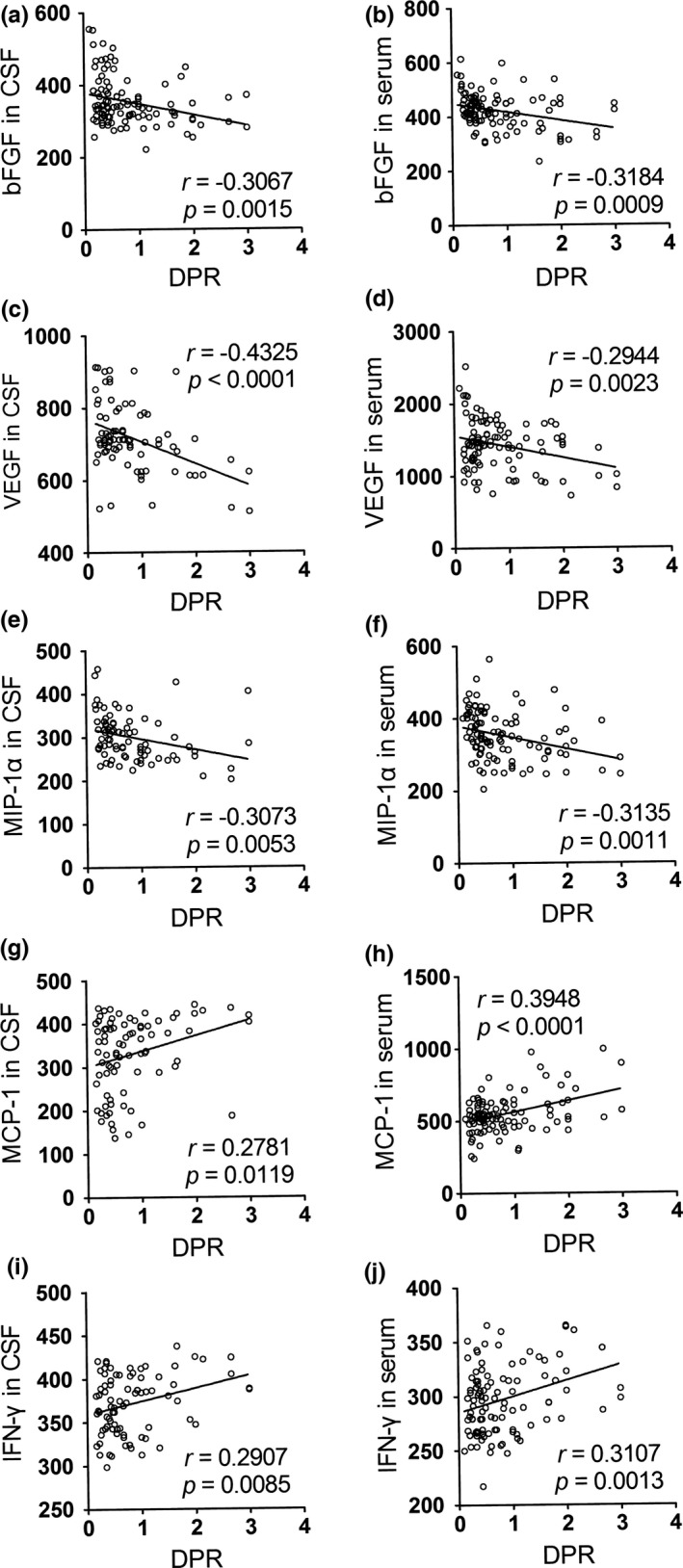
Correlations between the level of biomarkers in CSF/serum and DPR. (a) The level of bFGF in CSF was negatively correlated with DPR of ALS patients. (b) The correlation between the bFGF level in serum and DPR was negative. (c) The level of VEGF in CSF was negatively correlated with DPR of ALS patients. (d) The correlation between the VEGF level in serum and DPR was negative. (e) The level of MIP‐1α in CSF was negatively correlated with DPR of ALS patients. (f) The correlation between the MIP‐1α level in serum and DPR was negative. (g) The level of MCP‐1 in CSF was positively correlated with DPR of ALS patients. (h) The correlation between the MCP‐1 level in serum and DPR was positive. (i) The level of IFN‐γ in CSF was positively correlated with DPR of ALS patients. (j) The correlation between the IFN‐γ level in serum and DPR was positive

To further assess whether the changes in these factors had diagnostic value, we performed a series of correlation analyses between the factors and clinical parameters, including disease duration, ALSFRS‐r scores and DPR. We found that bFGF, VEGF, and MIP‐1α levels in the CSF and serum were positively correlated with disease duration (Table 3 and Figure 1), whereas opposing results were obtained when their correlation with DPR was analyzed (Table 5 and Figure 3). The levels of MCP‐1 in the CSF and serum were negatively correlated with the ALSFRS‐r scores of ALS patients (Table 4 and Figure 2); however, these levels showed a positive correlation with DPR (Table 5 and Figure 3). Moreover, in correlation analyses of DPR, IFN‐γ displayed a significant positive correlation in both the CSF and serum (Table 5 and Figure 3).

**Table 3:** Correlations between biomarker levels and disease duration in ALS patients

Marker	CSF		Serum	
	*r*	*p*	*r*	*p*
IL‐2	−0.1115	0.3215	0.0230	0.8159
IL‐6	−0.1621	0.1482	0.0187	0.8501
IL‐10	−0.0927	0.4103	−0.0051	0.9592
IL‐15	−0.0766	0.4965	−0.0735	0.4561
IL‐17	0.1761	0.1158	−0.0343	0.7282
G‐CSF	−0.0069	0.9516	−0.0625	0.5266
GM‐CSF	−0.0685	0.5436	−0.1154	0.2411
bFGF	0.3059	**0.0055**	0.3284	**0.0006**
VEGF	0.3387	**0.0020**	0.3099	**0.0013**
MIP‐1α	0.3106	**0.0048**	0.2911	**0.0026**
MIP‐1β	0.1745	0.1192	0.1403	0.1534
MCP‐1	0.0032	0.9770	−0.0979	0.3203
IFN‐γ	−0.0776	0.4909	−0.1141	0.2463

The bold values indicates that the results are statistically significant.

**Table 4:** Correlations between biomarker levels and ALSFRS‐r scores in ALS patients

Marker	CSF		Serum	
	*r*	*p*	*r*	*p*
IL‐2	−0.0564	0.6172	−0.1299	0.1865
IL‐6	0.1121	0.3191	−0.0380	0.7003
IL‐10	0.2026	0.0697	0.0369	0.7084
IL‐15	0.0185	0.8695	−0.0287	0.7716
IL‐17	−0.1862	0.0960	0.1594	0.1043
G‐CSF	0.0036	0.9748	−0.1108	0.2604
GM‐CSF	0.0936	0.4058	−0.0178	0.8567
bFGF	−0.0247	0.8268	−0.0186	0.8503
VEGF	−0.0567	0.6149	−0.0452	0.6473
MIP‐1α	0.0421	0.7093	0.0546	0.5803
MIP‐1β	−0.0101	0.9287	−0.0180	0.8554
MCP‐1	−0.2616	**0.0183**	−0.1999	**0.0409**
IFN‐γ	−0.1497	0.1823	−0.0785	0.4259

The bold values indicates that the results are statistically significant.

**Table 5:** Correlations between biomarker levels and DPR in ALS patients

Marker	CSF		Serum	
	*r*	*P*	*r*	*p*
IL‐2	0.1476	0.1884	0.0679	0.4912
IL‐6	0.1413	0.2082	0.0097	0.9221
IL‐10	−0.1361	0.2257	−0.0091	0.9262
IL‐15	0.0989	0.3796	0.0102	0.9177
IL‐17	0.0752	0.5049	−0.0360	0.7157
G‐CSF	0.1046	0.3528	0.1219	0.2154
GM‐CSF	−0.0396	0.7254	0.1557	0.1126
bFGF	−0.3067	**0.0015**	−0.3184	**0.0009**
VEGF	−0.4325	**<0.0001**	−0.2944	**0.0023**
MIP‐1α	−0.3073	**0.0053**	−0.3135	**0.0011**
MIP‐1β	−0.1888	0.0914	−0.1286	0.1910
MCP‐1	0.2781	**0.0119**	0.3948	**<0.0001**
IFN‐γ	0.2907	**0.0085**	0.3107	**0.0013**

The bold values indicates that the results are statistically significant.

### 3.3 Predictors of survival in ALS

After the aforementioned analysis, several factors have exhibited obvious connections with disease severity and progression. Thus, we asked whether these factors could predict survival using univariate and multivariate survival analyses. Among the 81 recruited ALS patients who provided both CSF and serum, 44 patients were deceased, and 37 (follow‐up period from 3 months to 60 months) were still alive at the time of last visit. In the univariate analysis, we analyzed the following factors to determine which of these were related to disease prognosis: gender; age; site of onset; BMI; duration; ALSFRS‐r; DPR and levels of bFGF, VEGF, MIP‐1α, MCP‐1, IFN‐γ in the CSF and serum. Subsequently, we used *p *<* *.2 to select covariates for the multivariate Cox proportional hazard model to adjust for the effects of the multivariate analysis. A better prognosis was observed with higher levels of bFGF in the CSF and VEGF in either the CSF or serum. Interestingly, survival analysis showed that patients with higher levels of IFN‐γ in the CSF had a shorter overall survival. A summary of the survival analysis is shown in Table 6.

**Table 6:** Analysis of the survival of ALS patients using univariate and multivariate Cox proportional hazards models

Variable	Univariate analysis			Multivariate analysis		
	HR	95% CI	*p*	HR	95% CI	*p*
Gender						
Female	1					
Male	1.204	0.629–2.301	0.575			
Age	1	0.973–1.028	0.988			
Site of onset						
Both	1					
Limb	1.225	0.292–5.137	0.781			
Bulbar	1.875	0.422–8.315	0.409			
BMI	0.917	0.841–0.999	0.049	0.976	0.887–1.074	0.615
Duration	0.987	0.972–1.002	0.095	0.992	0.964–1.021	0.580
ALSFRS‐r	0.974	0.938–1.011	0.165	0.970	0.898–1.046	0.426
DPR	2.092	1.473–2.972	<0.001	0.752	0.325–1.738	0.504
Serum bFGF	0.999	0.994–1.004	0.787			
CSF bFGF	0.983	0.975–0.991	<0.001	0.986	0.977–0.995	**0.002**
Serum VEGF	0.999	0.998–0.999	0.002	0.998	0.997–1.000	**0.016**
CSF VEGF	0.994	0.991–0.997	<0.001	0.994	0.990–0.999	**0.020**
Serum MIP‐1α	0.993	0.988–0.997	0.003	0.997	0.992–1.003	0.397
CSF MIP‐1α	0.996	0.990–1.002	0.234			
Serum MCP‐1	1.004	1.001–1.006	0.002	1.001	0.999–1.004	0.324
CSF MCP‐1	1.004	1.000–1.008	0.027	1.001	0.997–1.006	0.583
Serum IFN‐γ	1.006	0.996–1.015	0.243			
CSF IFN‐γ	1.024	1.014–1.035	<0.001	1.019	1.007–1.030	**0.001**

The bold values indicates that the results are statistically significant.

## 4 Discussion

In this study, we utilized ELISAs to identify a panel of serum and CSF factors for ALS, with five significant findings as follows: (i) A total of eight factors were obviously elevated in the CSF, and twelve factors were increased in serum; (ii) VEGF levels in either serum or the CSF positively correlated with disease duration, negatively correlated with DPR and could be a potential predictor for prognosis; (iii) Similar correlations were found with bFGF, except that only bFGF levels in the CSF showed significance in the survival analysis; (iv) MIP‐1α levels were positively associated with duration and negatively correlated with DPR, whereas MCP‐1 levels in both serum and the CSF were negatively correlated with ALDFRS‐r scores and had opposing relevance with DPR; and 5) A positive relationship with DPR was found with IFN‐γ, and a poor prognosis was associated with higher levels of IFN‐γ in the CSF. These factors, including cytokines, chemokines, and growth factors (which are involved with neuroinflammation and impaired neurotrophic support) are proposed to influence the pathogenesis of ALS.

Neuroinflammation plays an important role in disease progression of ALS. At regions of motor neuron injury, inflammation is a prominent pathological change and is indicated by microglial activation, astrogliosis, infiltration of monocytes and T cells, and the release of proinflammatory versus anti‐inflammatory cytokines (Zhao, Beers, & Appel, 2013). In our study, elevated interleukin levels comprising IL‐2, IL‐6, IL‐10, IL‐15, and IL‐17 were noted in the serum from ALS patients, whereas only IL‐15 and IL‐17 were clearly increased in the CSF. One possible explanation for the difference observed from the CSF and serum is that IL‐2, IL‐6, and IL‐10 might have a limited input in central nervous system (CNS) during the inflammatory process; as one previous study has shown, these cytokines are at similar levels in the CSF of ALS patients and control subjects (Tateishi et al., 2010). Furthermore, even IL‐2 and IL‐10 could not be detected in the CSF of ALS patients (Holmoy, Roos, & Kvale, 2006). In addition, no correlations were discovered between these interleukin levels and the clinical parameters in our analyses, although IL‐10 in serum was reported as a predictor of total disease duration (Su et al., 2013). These results may suggest that these interleukins were released erratically in response to the inflammation caused by ALS as systemic inflammation factors and have low susceptibility and specificity as biomarkers for ALS.

The increase in serum G‐CSF and serum GM‐CSF discovered in our research also weakly points to changes of neuroinflammation in ALS. G‐CSF is a traditional hematopoietic growth factor that was approved for granulocytopenia therapy. In addition to affecting the hematopoietic system, G‐CSF can exert anti‐inflammatory effects as well as promote neurogenesis in the CNS (Pitzer et al., 2008). However, GM‐CSF is regarded as a proinflammatory cytokine to facilitate proliferation and differentiation of inflammatory cells (Barbeito, Mesci, & Boillee, 2010). In our study, we could not detect any significant difference in the CSF between ALS patients and control subjects, nor was there any correlation with either disease progression or prognosis. Although elevated G‐CSF and GM‐CSF in the CSF were noted in several studies (Mitchell et al., 2009; Tateishi et al., 2010), their predictive and therapeutic values in ALS were also preliminarily evaluated (Su et al., 2013; Zhang et al., 2009). Differences in race or patient selection among the studies may be cause of these discrepancies. Further studies with a large‐scale population to investigate the exact changes and effects of G‐CSF in ALS are forthcoming.

Compared to unobvious alterations of the G‐CSF and GM‐CSF, three chemokines in our study emerged with strong differences between ALS patients and control subjects. Chemokines are a group of secreted proteins whose primary function is to induce inflammatory cell migration (Ramesh, MacLean, & Philipp, 2013). Interestingly, MIP‐1α and MCP‐1 showed inverse patterns with regard to the clinical parameters: MIP‐1α was positively associated with disease duration and negatively related with DPR, whereas MCP‐1 exhibited a negative correlation with disease severity and was positively correlated with DPR, suggesting that MIP‐1α and MCP‐1 interact with different chemokine signal pathways in neuroinflammation. MCP‐1, which is also known as CCL2, is considered to be a proinflammatory chemokine and believed to exert a detrimental effect by activating the CCR2 receptor in the inflammatory process (Jaerve & Muller, 2012). Nevertheless, CCR1 and CCR5 are receptors of MIP‐1α, which differ from those of MCP‐1 and may explain the differential effects between MIP‐1α and MCP‐1, although the complex relationship between chemokine and receptor is still vague. In the light of previous results demonstrating a correlation between MCP‐1 levels and disease severity (Tateishi et al., 2010), our study suggests that MCP‐1 has a predictive value for ALS progression and is possibly neurotoxic. In addition, similar conclusions were reached in our previous report that MIP‐1α is likely neuroprotective and possibly can monitor disease progression (Yang et al., 2016). Finally, MIP‐1β was noted as exerting an unimpressive influence in neuroinflammation as observed by elevation only in the CSF.

IFN‐γ is also critical to the neuroinflammatory process and has always been considered as a proinflammatory cytokine to promote the proliferation of cytotoxic microglia (Evans, Couch, Sibson, & Turner, 2013). Here, elevated IFN‐γ levels represented an active immune response in the CNS and periphery, which is in accordance with a previous report (Tateishi et al., 2010). Importantly, our analyses in correlation and survival implied that IFN‐γ is a potential biomarker that can distinguish ALS patients from control subjects, monitor progression and predict the prognosis of patients with ALS. In brief, the immune response is neither beneficial nor deleterious by definition but rather a double‐edged sword with different mediators under different conditions (Philips & Robberecht, 2011). Our study of cytokines above may provide insights into the neuroinflammatory aspect of ALS.

Apart from neuroinflammation, impaired neurotrophic support has also been long recognized as a hallmark of ALS. Trophic factors guide the generation and location of motor neurons; furthermore, they are involved in axon guidance and synapse formation (Tovar‐y‐Romo, Ramirez‐Jarquin, Lazo‐Gomez, & Tapia, 2014). We choose common neurotrophic factors, including bFGF and VEGF, and surveyed their differences between ALS patients and control subjects. VEGF is associated with angiogenesis and has recently been shown to exert neurotrophic effects on neurons (Keifer, O'Connor, & Boulis, 2014); a similar trophic effect was also found with bFGF (Chen, Cai, Shen, Cai, & Lei, 2014). We measured increased bFGF and VEGF levels in both the CSF and serum and noted positive correlations with disease duration but negative associations with DPR. Additionally, higher levels of bFGF and VEGF indicated better prognosis as shown in our multivariate survival analysis. Our research confirms previous results, elucidating that increased bFGF and VEGF levels is potentially a reflexive neuroprotective mechanism in ALS (Mitchell et al., 2009).

Currently, a diagnosis of ALS is primarily based on medical history, clinical examination, electrophysiological results, and the exclusion of similar disorders. Therefore, finding reliable biomarkers that could provide accurate information regarding the onset and progression of ALS in clinical practice and trials is urgently necessary. The past year has seen progress in a range of areas, multiple candidate biomarkers have emerged, specifically from neurochemical analysis of biofluids (Chen et al., 2016; Xu, Henderson, David, & McCombe, 2016), neuroimaging biomarkers (Cardenas‐Blanco et al., 2016); and neurophysiological techniques (Neuwirth et al., 2016). This study reported comprehensive results focusing on a panel of biomarkers both in the CSF and serum, which provided ample screening of potential predictors used a novel multivariate model. However, several limitations of this study must be noted. First, there was a small sample number chosen in the Cox proportional hazard model, so we describe the results as preliminary to set the stage for large‐scale prognostic biomarkers analyses. Moreover, this is a retrospective design that used samples collected at a single time point rather than measures the factor levels during disease course. Therefore, a longitudinal study that obtains serial samples from ALS patients to observe the dynamic alterations of these factors is necessary.

## 5 Conclusions

In conclusion, we identified a panel of promising biomarker candidates of ALS that may be useful in clinical practice and therapeutic trials and that offer diagnostic, prognostic or monitoring potential to help elucidate the pathogenic mechanism of ALS. Moreover, CSF is more appropriate for biomarker discovery than serum because of its proximity to the affected regions exhibiting ALS‐induced motor neuron death. More large‐scale studies with detailed longitudinal follow‐up will be necessary to further evaluate the value of these biomarkers.

## Disclosures

The authors declare no financial or other conflict of interests.
